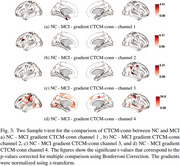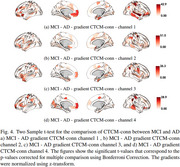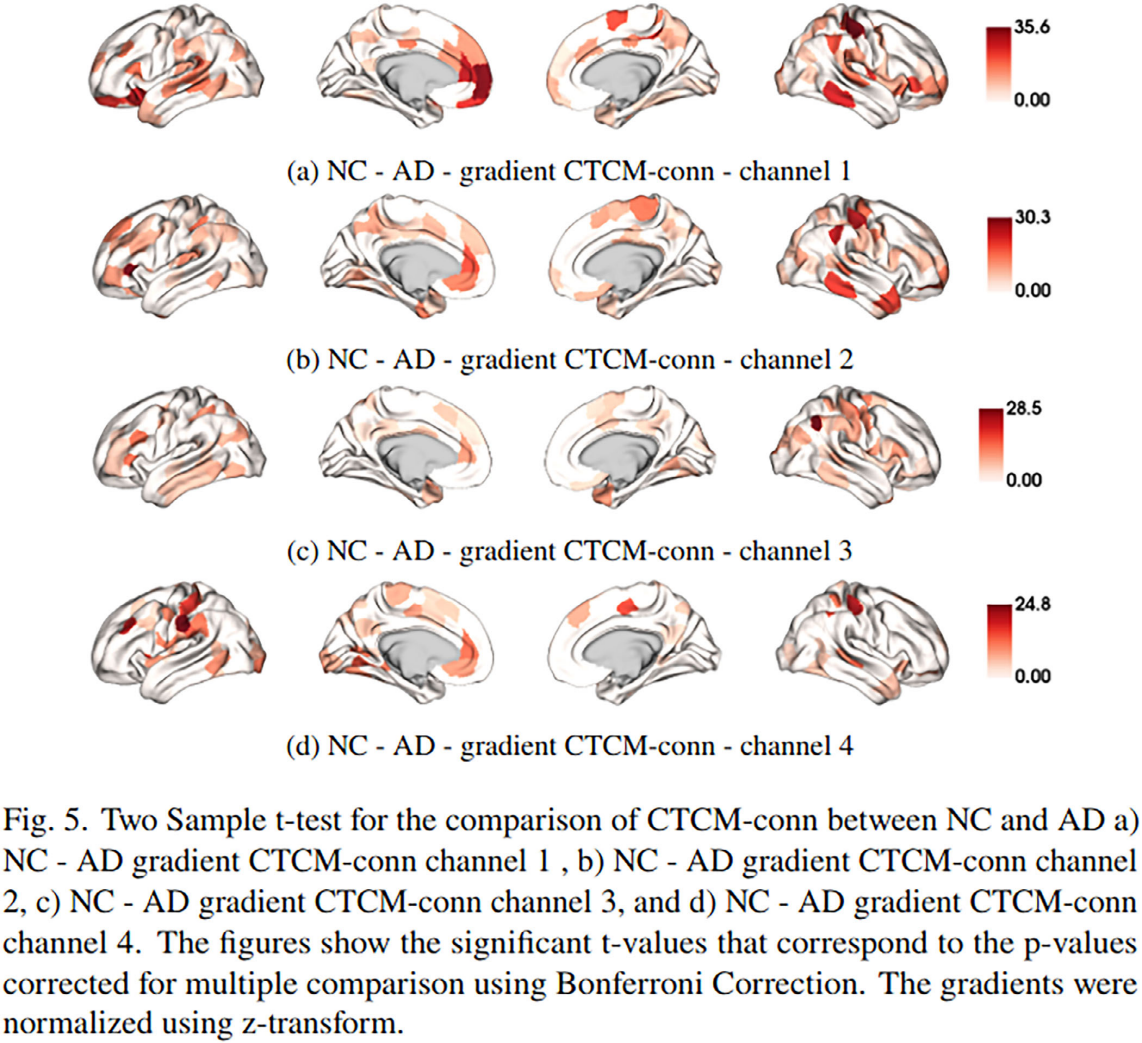# Cross Time Coherence Mapping Gradient Analysis for Alzheimer's Disease Assessment

**DOI:** 10.1002/alz70856_104151

**Published:** 2025-12-26

**Authors:** Aldo Camargo, Ze Wang

**Affiliations:** ^1^ University of Maryland of Baltimore, Baltimore, MD, USA

## Abstract

**Background:**

Dysconnectivity has been consistently identified in Alzheimer's Disease (AD) through inter‐regional correlation analyses, yet cross‐time coherence mapping (CTCM) for understanding information exchange remains underexplored. This study utilized resting‐state fMRI (rsfMRI) to compute CTCM connectivity (CTCM_conn) and gradient analysis to investigate inter‐relationships among 300 brain regions in normal controls (NC), mild cognitive impairment (MCI), and AD groups.

**Method:**

Forty NC, 38 MCI, and 40 AD participants were analyzed. Preprocessing steps (motion correction, slice timing correction, normalization, and artifact removal) were performed using SPM12 and FSL. Time series were extracted from 300 Schaefer atlas seeds, and 300×300 CTCM_conn matrices were computed for each subject. Gradients were calculated using the BrainSpace toolbox across four CTCM_conn channels. Population‐level mean matrices and gradients were computed for each group, and individual gradients were aligned for comparison. Statistical differences were tested with two‐sample t‐tests and Bonferroni correction. Machine learning models, incorporating gradients, age, and gender, were evaluated for classifying NC, MCI, and AD, with robustness tested via 1,000 bootstrapped samples.

**Result:**

Significant gradient differences were identified:

**NC vs. MCI**: Prominent differences in the visual cortex and Salience/Ventral Attention (SalVentAttn) networks.

**MCI vs. AD**: Differences in the visual, sensorimotor (SomMot), SalVentAttn, Default Mode (Default_PFC and Default_Temp), and dorsal attention (DorsAttn) networks.

**NC vs. AD**: Differences in the SomMot, DorsAttn, Limbic (Limbic_OFC and Limbic_TempPole), and Default_PFC networks.

Logistic regression outperformed other models using the original data, and after bootstrapping, most models achieved >90% accuracy.

**Conclusion:**

These findings reveal significant alterations in CTCM gradients across NC, MCI, and AD, reflecting the progressive dysconnectivity in AD. Key networks, including the Default Mode, Dorsal Attention, Salience, and Limbic networks, exhibited marked differences. Combining CTCM gradients with demographic features demonstrates strong potential as a biomarker for AD progression and classification, offering new insights for diagnosis and understanding disease mechanisms.